# Transactions of the Maine Homœopathic Medical Society

**Published:** 1890-12

**Authors:** 


TRANSACTIONS OF THE MAINE HOMŒOPATHIC MEDICAL SOCIETY at its Twenty-fourth Annual Meeting held at Bangor, June 3d, 1890. Portland, Me.: Brown, Thurston Co., 1890.

This is a volume of one hundred and thirty-six pages, containing the discussions and papers read at the last annual meeting. The papers are interesting, but we regret to find that so many of them advocate the treatment of the old school, and several others teach alternation of remedies and external applications. This kind of practice and teaching does not advance the cause of Homœopathy. On the contrary, it maintains a state of confusion and doubt in the minds of the majority that must cause Homœopathy to stand still or else retrograde. W. M. J.

